# Short-term anti-vascular endothelial growth factor treatment elicits vasculogenic mimicry formation of tumors to accelerate metastasis

**DOI:** 10.1186/1756-9966-31-16

**Published:** 2012-02-23

**Authors:** Yuan Xu, Qin Li, Xiao-Yu Li, Qiu-Ya Yang, Wei-Wei Xu, Gao-Lin Liu

**Affiliations:** 1 Department of Pharmacy, Shanghai First People's Hospital, School of medicine, Shanghai Jiao Tong University, No.100 Haining Road, Shanghai, 200080, China; 2 Department of Orthopaedics, Yangzhou First People's Hospital, Jiangsu, China

**Keywords:** Antiangiogenic therapies, Metastasis, Hypoxia, Vasculogenic mimicry

## Abstract

**Background:**

Antiangiogenic therapy is one of the most significant advances in anticancer treatment. The benefits of antiangiogenic therapies of late-stage cancers have been investigated but are still too limited.

**Methods:**

We used an ovarian cancer model to test the effect of short-term bevacizumab treatment on metastasis as measured by bioluminescence. Western blotting and CD34-PAS dual staining were performed to assess hypoxia-inducible transcription factor-1α (HIF-1α) expression and vasculogenic mimicry(VM) formation. Cell viability was examined by a CCK8 assay.

**Results:**

Bevacizumab demonstrated antitumor effects in models of ovarian cancer, but also accelerated metastasis together, with marked hypoxia and VM formation in mice receiving short-term therapy. Bevacizumab treatment did not affect SKOV3 cell viability and the amount of VM in three-dimensional culture.

**Conclusion:**

These results suggest that antiangiogenic therapy may potentially influence the progression of metastatic disease, which has been linked to the hypoxic response and VM formation.

## Background

Tumors can grow to a maximum diameter of between 1 and 2 mm before their metabolic demands are restricted due to the diffusion limit of oxygen and lack of essential nutrients. To exceed this size or spread to other organs, tumors require an independent blood supply. In the 1970s, Folkman et al was the first to propose the concept of antiangiogenesis as a therapeutic approach to treat solid tumors [1]. Targeting the blood supply by inhibiting the formation of blood vessel will lead to tumor growth arrest. Numerous angiogenesis inhibitors have been therapeutically used in both preclinical and clinical settings [2]. Vascular endothelial growth factor (VEGF) receptor tyrosine kinase inhibitors and a VEGF-neutralizing antibody have been clinically validated to target VEGF or its receptors as an anticancer treatment. However, a number of limitations are observed in current antiangiogenic therapies. Many clinical benefits are short-lived, and enduring clinical responses are rare. While numerous trials have shown an increase in survival after patients are treated with antiangiogenic therapy, the increase for many was only a matter of months [3]. Moreover, single-agent use of antiangiogenesis appears to be insufficient to improve patient survival [4]. While any improvement in overall survival for patients should be regarded as advancement, it is importart to understand why such clinical improvements are sometimes transitory so that new therapies result in more enduring benefits.

One explanation for these limitations is a potential link between antiangiogenic therapy and increased metastasis [5]. In RIP-Tag2 mice treated with the VEGF receptor 2-inhibitor DC101, although tumors were smaller, they showed significantly more invasive and malignant phenotypes, with most showing wide fronts of invasion into urrounding acinar tissues [6]. Rodents treated with an anti-VEGF antibody showing a striking increase in the number and total area of small satellite tumors compared with those that had not received antiangiogenic therapy, and tumor cells often had migrated over long distances [7,8]. Together, these results suggest that antiangiogenic therapy may influence the progression of metastatic disease. To understand the reasons for these observations and to enable enduring benefits of antiangiogenic therapies, we examined the effect of a VEGF-neutralizing antibody on metastasis in mice after short-term administration. Furthermore, the hypoxic response and vasculogenic mimicry (VM) formation were assessed in this study.

## Materials

### Antibodies

For western blotting and histopathological analyses, a mouse anti-HIF-1α monoclonal antibody was purchased from Novus Biologicals (Littleton, CO, USA), CD34 monoclonal antibody from Abgent (San Diego, CA, USA).

### Cell lines

The human ovarian cancer cell line SKOV3 was purchased from the ATCC and transfected with a luciferase-expressing lentivirus containing an independent open-reading frame of GFP. After 72 hours, cells were examined by fluorescence microscopy to confirm infection. Luciferase expression was determined using luciferin and an in vivo imaging system (Xenogen). Cells were maintained in RPMI-1640 medium supplemented with 10% heatinactivated fetal bovine serum (Gibco Invitrogen Corp), and incubated at 37°C in a humidified atmosphere containing 5% CO_2_.

### Three-dimensional(3D) cultures

Matrigel (BD Biosciences) was placed dropwise onto glass coverslips in 12-well culture plates and allowed to polymerize for 30 min at 37°C. SKOV3 cells were then seeded onto the 3D matrix in complete medium.

### Animal models

SKOV3^LUC+ ^cells (1.2 × 10^6 ^cells) were directly injected into the tail vein of 6- 8-week-old female nude mice. Forty mice were assigned into four groups(A, B, C and D). Group A was treated with phosphate-buffered saline (PBS) bi-weekly for 3 weeks. Group B was treated with 40 mg/kg bevacizumab bi-weekly for 3 weeks. Group C was treated with 3 mg/kg cisplatin weekly for 3 weeks. Group D was treated with both bevacizumab bi-weekly and cisplatin weekly for 3 weeks. Bevacizumab and cisplatin were administered intraperitoneally. Body weight was measured and recorded weekly. Metastatic disease progression in SKOV3^LUC+ ^tumor-bearing mice was monitored. Before mice were anesthetized with Forane, an aqueous solution of luciferin (150 mg/kg) was intraperitoneally injected at 10 min prior to imaging. Mice were placed into the light-tight chamber of a CCD camera system (Xenogen), and photons emitted from luciferase-expressing cells within mice were quantified for 1 min, using the software program living. Four weeks after initial treatment, all mice were sacrificed to assess the effects of drug treatments. All procedures involving mice complied with the Guide for the Care and Use of Laboratory Animals (National Institutes of Health).

### Western blotting

The tissues were homogenized in 0.5 ml Hepes (50 mM, pH 7.5) containing 100 mM NaCl, 1 mM CaCl_2_, 1 mM dithiothreitol, 1% ethylene glycol-bis(aminoethyl ether)-tetraacetic acid 1% Triton X- 100 and proteinase inhibitors. Protein extracts were kept in ice for 30 min and then centrifuged at 14,000 g at 4°C for 30 min. Protein concentrations were determined using a bicinchoninic acid protein assay reagent kit. Protein samples (20 mg) were mixed with equal volumes of loading buffer (20% glycerol, 4% sodium dodecyl sulfate, and 100 mM Tris-HCl, pH 6.8) and then boiled for 5 min in the presence of β-mercaptoethanol. Proteins were separated in 8% sodium dodecyl sulfate-polyacrylamide gels at 100 V for 2 h and then electrotransferred to nitrocellulose membranes at 270 mA for 2 h. Membranes were blocked with 5% non-fat dry milk in PBS with 0.1% Tween 20 for 1 h at room temperature. Then, membranes were incubated with anti- HIF-1α (1:500) overnight at 4°C and finally with a horseradish peroxidase-conjugated anti-mouse IgG for 1 h at room temperature after washing with TBS containing 0.1% Tween 20. Proteins were visualized by enhanced chemiluminescence reagents after washing. Protein expression was semi-quantified using an image analysis system.

### CD34-PAS dual staining

Four micrometer paraffin sections were routinely deparaffinized and dehydrated. First, CD34 immunohistochemical staining was applied to the sections. Endogenous peroxidase activity was blocked with 3% hydrogen peroxide in 50% methanol for 10 min at room temperature. Sections were rehydrated and washed with PBS and then pretreated with citrate buffer (0.01 M citric acid, pH 6.0) for 20 min at 100°C in a microwave oven. Non-specific binding sites were blocked with 2% normal goat serum in PBS for 20 min at 37°C. Sections were then incubated overnight at 4°C with anti-CD34 at a 1:200 dilution. Then, sections were rinsed with PBS and incubated with biotinylated goat anti-mouse IgG for 20 min at 37°C, followed by incubation with 3,3'-diaminobenzidine(DAB) chromogen for 10 min at room temperature. Sections were then rinsed with water for 1 min to stop the DAB-staining reaction. Formalin and melanin granules were then removed using the methods mentioned above. Finally, sections were treated with 0.5% periodic acid solution for 10 min and rinsed with distilled water for 2-3 min. In a dark chamber, sections were treated with Schiff solution for 15-30 min. After rinsing with distilled water, sections were counterstained with hematoxylin [9].

### Cell counting kit 8 (CCK8) assay

Cell viability was measured by conversion of Dojindo's highly water-soluble tetrazolium salt WST-8 to a yellow-colored watersoluble formazan (CCK8 assay). The amount of formazan dye generated by the activity of mitochondrial dehydrogenases in cells is directly proportional to the number of living cells. CCK8 is more sensitive than the 3-(4,5-dimethylthiazol-2-yl)-2, 5-diphenyltetrazolium bromide assay [10]. SKOV3 cells were trypsinized and seeded at 5 × 10^3 ^cells/well in 96 well plates in 3D cultures. After 24 h, various concentrations of bevacizumab were added, followed by incubation for another 48 h. Then, 10 μL CCK8 (Sigma, USA) solution in PBS was added to each well. Plates were incubated for an additional 2 h. The optical density of each well was measured using a microculture plate reader at a 490 nm wavelength.

### Statistical analysis

All results were evaluated using the SPSS 13.0 statistical software package. Data were analyzed using one-way ANOVA. Results were expressed as the mean ± standard deviation, and P < 0.05 was considered statistically significant.

## Results

### Increased metastasis after short-term treatment with the angiogenesis Inhibitor bevacizumab

**Figure 1:**
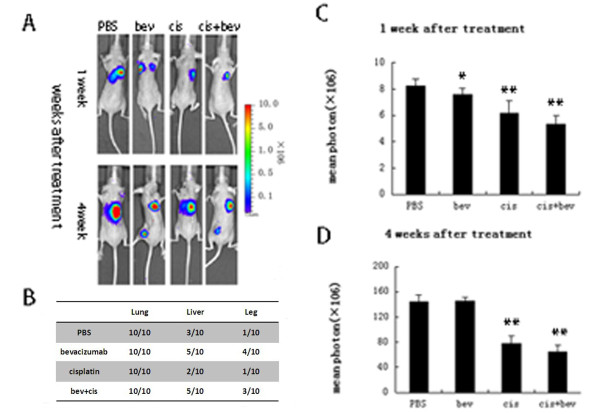
**Increased metastasis after short-term treatment with bevacizumab**. Forty mice were assigned into four groups (PBS, bevacizumab, cisplatin and bevacizumab + cisplatin). Mean photon counts of each group were quantified. (**A**) Tumor growth and metastasis were monitored by bioluminescence at 1 and 4 weeks after treatment. A representative experiment is shown. (**B**) Short-term bevacizumab treatment resulted in accelerated extrapulmonary metastasis at 4 weeks after treatment. Extrapulmonary metastases were found in the livers and legs. The ratio of metastasis of each group was measured. (**C**) Quantification of bioluminescence showed inhibited tumor growth in bevacizumab and/or cisplatin treatment groups at 1 week after treatment, compared with that of the PBS group. Bevacizumab + cisplatin treatment inhibited tumor growth, compared with that of cisplatin at 1 week after treatment. (**D**) Quantification of bioluminescence showed no significant difference in tumor growth between bevacizumab and PBS groups 4 weeks after treatment. Bevacizumab + cisplatin treatment inhibited tumor growth compared with that of cisplatin at 4 weeks after treatment. *P < 0.05, **P < 0.01.

In our study, a model of metastasis was used to test the effect of short-term bevacizumab treatment. SKOV3^LUC+ ^cells expressing luciferase were directly injected into the tail vein of female nude mice and then received bevacizumab and/or cisplatin treatment for 3 weeks. Forty mice were equally divided into four groups at random (PBS, bevacizumab, cisplatin and bevacizumab + cisplatin groups). Tumor growth and metastasis were monitored by bioluminescence at 1 and 4 weeks after treatment. Mean photon counts of each group were quantified, and the ratio of metastasis was measured. The pulmonary metastasis rate was 100%. Tumor growth delay was observed at 1 week after bevacizumab and/or cisplatin treatments, without extrapulmonary metastasis. Short-term bevacizumab treatment resulted in accelerated extrapulmonary metastasis at 4 weeks after treatment. Extrapulmonary metastases were found in livers and legs. Cisplatin and bevacizumab + cisplatin treatment inhibited tumor growth, compared with that of PBS treatment.. While no significant difference in tumor growth was observed between bevacizumab and control groups (Figure 1).

### Hypoxia is implicated in the adaptive response

**Figure 2:**
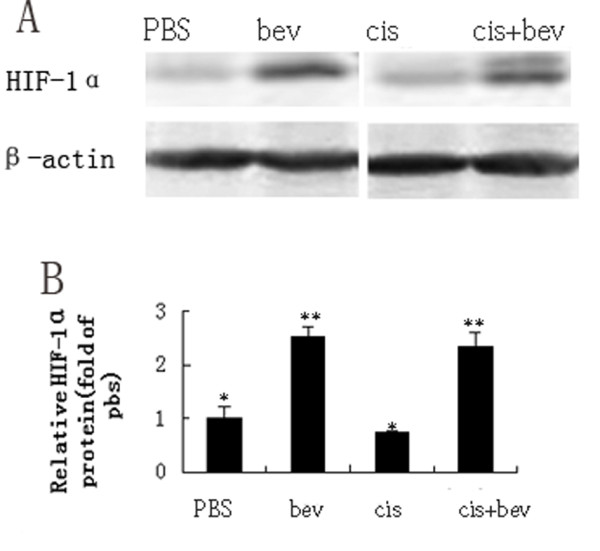
**Hypoxia is implicated in the adaptive response after short-term bevacizumab treatment**. Expression of HIF-1α in pulmonary tumor nodules of the four groups. (**A**) A representative western blot is shown. β-actin was used as a loading control. (**B**) While most tumors showed little expression of HIF-1α protein in PBS and cisplatin groups, mice that received bevacizumab and bevacizumab + cisplatin therapy showed a markedly increased level of HIF-1α expression.. *P < 0.05, **P < 0.01.

To gain an insight into possible molecular mechanisms of the increased metastasis, we determined whether hypoxia development was concomitant with metastasis. Mice were assigned into four groups (PBS, bevacizumab, cisplatin and bevacizumab + cisplatin) and received bevacizumab and/or cisplatin treatments for 3 weeks. Four weeks after initial treatment, five mice from each group were sacrificed for examination. Expression of HIF-1α in pulmonary tumor nodules was analyzed by western blotting. In PBS and cisplatin groups, most tumors showed little hypoxia. In contrast, mice that received bevacizumab and bevacizumab + cisplatin therapy showed a markedly increased level of HIF-1α expression (Figure 2). Differences in HIF-1α protein levels in each group were considered statistically significant.

### Anti-VEGF treatment also induces increased VM

**Figure 3:**
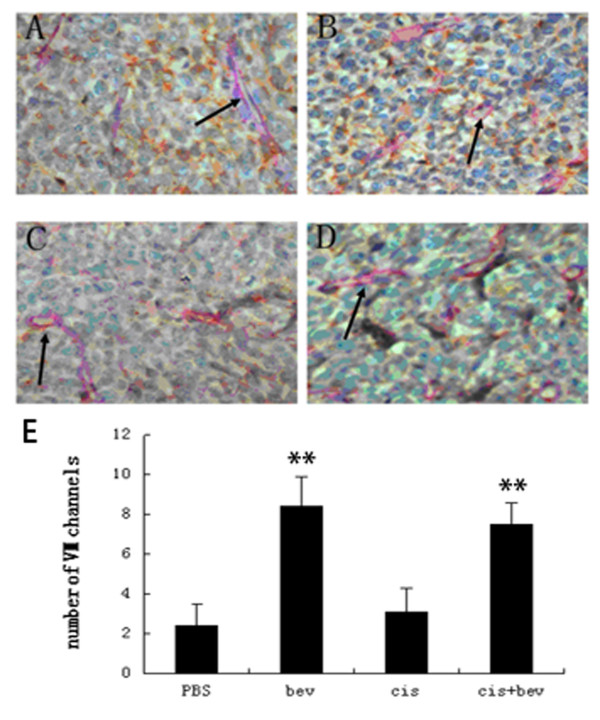
**Anti-VEGF treatment induces increased VM**. Comparison of VM channels in mice with various treatments. VM channels were positive for PAS staining and negative for CD34 staining in sections (arrow, ×400). (**A**) PBS (**B**) bevacizumab (**C**) cisplatinp and(**D**) bevacizumab + cisplatin groups. (**E**) Comparison of VM channels in A, B, C and D. Tumors in the bevacizumab group formed more VM channels than that of PBS and cisplatin, and bevacizumab + cisplatin groups. **P < 0.01.

The definition of VM is that tumor cells mimic endothelial cells and form vasculogenic networks. CD34-PAS double staining was used to distinguish VM and endothelial-dependent vessels. CD34 is a marker of endothelial cells, and the basement membrane is positive for PAS. Therefore, we counted PAS-positive and CD34-negative vessels for indicate. Mice were assigned into four groups (PBS, bevacizumab, cisplatin and bevacizumab + cisplatin) that received bevacizumab and/or cisplatin treatments for 3 weeks. Four weeks after initial treatment, five mice from each group were sacrificed for examination. Tumors in the bevacizumab group formed more VM channels than those of PBS and cisplatin, and bevacizumab + cisplatin groups (Figure 3).

### In vitro experiment demonstrating the effect of bevacizumab on VM

**Figure 4:**
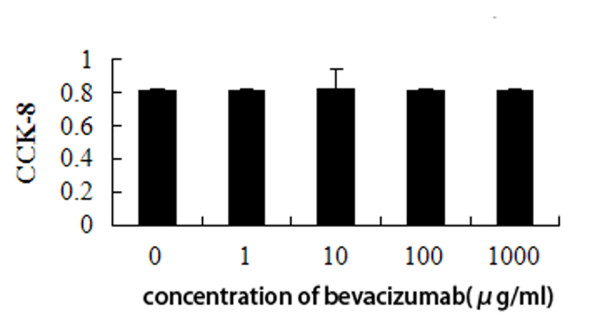
**Bevacizumab treatment did not affect SKOV3 cell viability**. Bevacizumab treatment (0, 1, 10, 100 and 1000 μg/ml) does not affect SKOV3 cell viability in 3D culture. There were no statistically significant difference (*P *> 0.05).

**Figure 5:**
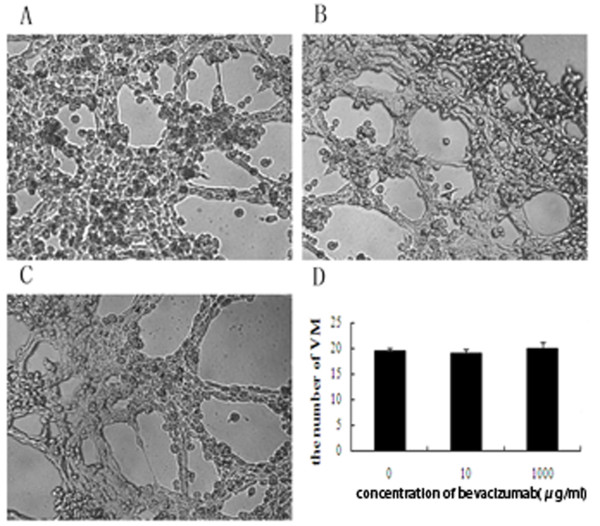
**Bevacizumab treatment did not affect the number of tubules**. The effect of bevacizumab (0, 10 and 1000 μg/ml) on the formation of VM channels (× 100). (**A**) Bevacizumab at 0/(**B**) 10/(**C**) 1000 μg/ml. (**D**) Bevacizumab treatment did not affect the number of tubules (*P *> 0.05).

SKOV3 cells were cultured in 3D culture, which formed VM channels. Then, we compared the cell viability and the ability to form VM in 3D culture after treatment with bevacizumab (0, 1, 10, 100 and 1000 μg/ml)for up to 48 h. Cell viability was examined by a CCK8 assay. Bevacizumab treatment did not affect SKOV3 cell viability and the number of tubules (Figures 4 and 5).

## Discussion

Antiangiogenic therapy is one of the most significant advances in cancer treatment. Its clinical value has been investigated, but is still too limited. A number of recent clinical and preclinical observations have been reported. In a neoadjuvant phase II trial of advanced epithelial ovarian cancer patients treated with the combinational therapy of carboplatin/paclitaxel with the angiogenesis inhibitor sorafenib, Pölcher M et al. reported that progressive disease was diagnosed in two patients out of four, and surgical exploration showed an increased number of peritoneal tumor implants [11]. Furthermore, after short-term treatment, varous forms of antiangiogenic therapy can lead to increased metastasis in mouse models of multiple tumor types [12,13]. Thus, there is a strong need to improve treatment strategies and to better understand the mechanisms of failure that hinder targeted antiangiogenic therapies. Here, we address the effect of short-term bevacizumab treatment using ovarian cancer xenografts. The data show that short-term bevacizumab treatment induces a reduction in tumor growth and an increase in distant tumor metastasis as measured by bioluminescence. Importantly, similar results were obtained when nu/nu mice were treated with bevacizumab + cisplatin and cisplatin alone. It should be noted that in mouse models of ovarian cancer, antiangiogenic therapy can elicit an adaptive response involving increased dissemination and the emergence of distant metastasis.

To investigate this metastatic "conditioning" effect, a better understanding of the biological effects of anti-VEGF treatment is required. Antiangiogenic therapy inhibits the development of new blood vessels, i.e. angiogenesis, resulting in reduced perfusion and an increase in intratumoral hypoxia, which may indirectly affect the tumor cells by inducing a more invasive phenotype in response to hypoxia, leading to increased invasion and metastatic dissemination [14,15]. HIF-1α is a main regulator of the transcriptional response of cancer cells to hypoxia. By analyzing HIF-1α expression using western blotting we showed that treatment with bevacizumab increases intratumoral hypoxia in metastasis models of ovarian cancer. While most tumors showed little or no expression of HIF-1α protein in groups without bevacizumab treatment, HIF-1α expression markedly increased both in bevacizumab and bevacizumab + cisplatin groups. In summary, short-term bevacizumab treatment results in increased of HIF-1α expression. Interestingly, HIF-1α regulates genes that are involved in angiogenesis, cell survival, invasion and metastasis [16]. Therefore, downstream pathways of HIF-1α gene may contribute to metastatic phenotypes.

Current antiangiogenic strategies are mainly directed against tumor endothelial cells. However, tumours do not only rely on host blood vessels for nourishment, they can also form their own vasculature. The term "VM" has been used to describe the manner in which tumor cells mimic endothelial cells to form vasculogenic networks. VM has been described in ovarian cancer and some other highly aggressive tumors such as melanoma, prostatic carcinoma, breast cancer, soft tissue sarcomas and lung cancer [17-22]. The presence of VM correlates to an increased risk of metastasis and poor clinical outcome [23-26]. Several key molecules, including VE-cadherin, matrix metalloproteinases, laminin-5 γ2 chain and EphA2, have been implicated in VM. Moreover, the tumor microenvironment, including hypoxia, ischemia and acidosis, plays a major role in trans-endothelial differentiation of aggressive tumor cells [27-30]. In the hypoxic microenvironment, melanoma cells increase HIF-1α expression and induce the formation of VM channels to acquire an adequate blood supply [31]. In 3D culture, bevacizumab treatment for up to 48 h did not affect SKOV3 cell viability and the ability to form VM. Moreover, our data showed more VM channels in short-term bevacizumab treatment groups than those in control groups. This feature suggests that VM channels, which cannot be inhibited by bevacizumab, may satisfy the vascular requirements of ovarian cancer growth, invasion and metastasis during hypoxia. Thus, the increased of VM formation as a result of bevacizumab-induced hypoxia may increase dissemination and the emergence of distant metastasis. These findings offer a possible explanation for why antiangiogenesis only shows transitory clinical benefits.

## Conclusions

VEGF inhibition causes hypoxia, induces HIF-1α expression and the formation of VM, which may be associated with tumor invasion and metastasis. Antiangiogenesis inhibits endothelium-dependent vessels, and then causes hypoxia in tumors. To compensate for tumor hypoxia, VM may increase to maintain the tumor blood supply and provide a convenient route for tumor metastasis. Another question raised by our results is whether other mechanisms linked to the hypoxic response could contribute to the metastatic phenotype, such as angiogenesis, growth factor signaling and apoptosis. The relationship between antiangiogenic therapy and metastasis remains to be determined and is an important topic for future research. Further study may provide additional drug targets, resulting in adjuvant therapies that can enhance the clinical benefits of antiangiogenic treatment.

## Competing interests

The authors declare that they have no competing interests.

## Authors' contributions

XY carried out the design of the experiments, performed most of experiments and drafted the manuscript. LQ participated in the design of the experiments, western blot and cell culture. LXY participated in statistical analysis and interpretation. YQY participated in animal experiments. XWW participated in the statistical analysis and helped drafting the manuscript. LGL participated in the design of the experiments and helped drafting the manuscript. All authors read and approved the final manuscript.
